# Longitudinal Associations Between Materialism and Problematic Smartphone Use in Adolescence: Within- and Between-Person Effects

**DOI:** 10.3390/bs16010150

**Published:** 2026-01-21

**Authors:** Xinran Dai, Huanlei Wang, Xiaoxiong Lai, Shunsen Huang, Xinmei Zhao, Yun Wang

**Affiliations:** 1 State Key Laboratory of Cognitive Neuroscience and Learning, Beijing Normal University, Beijing 100875, China; daixinran@mail.bnu.edu.cn (X.D.); wanghuanlei@mail.bnu.edu.cn (H.W.); zhaoxm@mail.bnu.edu.cn (X.Z.); 2 Chengdu Shisunjie Primary School, Chengdu 610031, China; 3 Institute of Digital Education, China National Academy of Educational Sciences, Beijing 100088, China; laixx@cnaes.edu.cn; 4 Institute of Sociology, Chinese Academy of Social Sciences, Beijing 100732, China; 5 Guangdong Experimental High School, Guangzhou 510000, China

**Keywords:** materialism, problematic smartphone use, random intercept cross-lagged panel model, between-person effect, within-person effect

## Abstract

Although there are theoretically expected associations between problematic smartphone use (PSU) and materialism, there is a lack of research that examines these associations using a longitudinal design, focusing on both within-person and between-person effects. Clarifying this relationship may inform interventions for these related conditions. Accordingly, data from three annual waves collected from a substantial group of Chinese adolescents (*N* = 3029, *M*_age_ = 12.26 ± 2.36, male: 50.00%) were used to assess within-person and between-person effects in the association between PSU and materialism. Traditional cross-lagged panel models were utilized to analyze the data, which consistently showed reciprocal positive associations between PSU and materialism across all waves. In contrast, the random intercept cross-lagged panel model revealed that PSU and materialism exhibited reciprocal associations over time at the between-person level. However, no significant cross-lagged linkage was observed between PSU and materialism at the within-person level. These findings enhance our understanding of the temporal dynamic relationship between PSU and materialism and underscore the necessity to disaggregate within-person and between-person effects to elucidate the nature of the longitudinal associations between PSU and materialism. The study also has implications for theoretical and practical understanding.

## 1. Introduction

Smartphones are increasingly used worldwide and have become an indispensable part of people’s lives (DataReportal, 2024). According to the report of internet usage released by the China Internet Network Information Center, smartphones are the most commonly utilized smart devices by adolescents in China (CNNIC, 2023, 2025). However, due to physiological and psychological immaturity, adolescents might struggle to manage their smartphone usage effectively (B. Li et al., 2023), and hence develop problematic smartphone use (PSU). While often termed “smartphone addiction” in earlier literature due to its symptomological similarities with behavioral addictions (e.g., withdrawal, tolerance), recent scholarship argues that the construct is conceptually distinct and should be distinguished from clinical pathology (Panova & Carbonell, 2018). Therefore, consistent with prevailing views, we adopt the term PSU to describe a maladaptive pattern of use characterized by a persistent urge to utilize a smartphone uncontrollably, resulting in disruptions to daily activities (Busch & McCarthy, 2021; Horwood & Anglim, 2018). Studies have revealed that teenagers exhibiting heightened levels of PSU frequently struggle with serious mental health issues, face difficulties in achieving academic success, and experience unpleasant social interactions (Elhai et al., 2017; Samaha & Hawi, 2016; Seo et al., 2016). 

Materialism is a system of beliefs that accentuates the significance of gaining and owning material personal assets throughout an individual’s life (Richins & Dawson, 1992). Materialists are prone to hold the belief that obtaining material possessions is essential for attaining their primary life goals or desired states of being (Kasser, 2016). With the rapid development of the social economy, materialistic values are becoming increasingly popular, especially among young people (Islam et al., 2017). This has raised global concerns about the increasing materialism among the younger generation, as it is believed to have detrimental effects on adolescents in the long run (Islam et al., 2017; Nagpaul & Pang, 2017; Schaefer et al., 2004). According to empirical studies, teenagers who exhibit elevated levels of materialism tend to have lower-quality interpersonal relationships, worse academic motivation and performance, and lower life satisfaction and well-being (Dittmar et al., 2014; J. Jiang et al., 2016a; Ku et al., 2014; Pieters, 2013).

Both theoretical and empirical evidence suggests that PSU is associated with an increased level of materialism (Sharif & Khanekharab, 2017; Wang et al., 2020a, 2020b) and that materialism may also confer susceptibility to the development of PSU (Donnelly et al., 2016; Gentina & Rowe, 2020; Kardefelt-Winther, 2014; Katz et al., 1973; Lee et al., 2018; Long et al., 2021; Ouyang et al., 2020). However, existing research on the associations between PSU and materialism has generally employed a cross-sectional design, posing challenges in making accurate inferences about the existence and magnitude of the potential reciprocal associations between them. Additionally, few existing studies have separately explored the within- and between-person effects between PSU and materialism, both of which can be valid and have their own unique theoretical and practical implications (Curran & Bauer, 2011). Therefore, using a longitudinal design, this study fits both traditional cross-lagged panel models (i.e., CLPM, a common analytic approach for testing the temporal dynamic associations between variables) and a random intercept cross-lagged panel model (i.e., RI-CLPM, a relatively advanced statistical method that can separate these two effects) and we compared the results to test the time dynamics to offer intervention methods for these potential conditions. 

## 2. Theoretical and Empirical Background

### 2.1. The Links Between PSU and Materialism: Theoretical and Empirical Evidence

#### 2.1.1. PSU as a Possible Antecedent of Materialism

Drawing on social learning theory (Bandura, 1962, 1969), prior research indicates that adolescents may emulate the luxurious lifestyles presented in smartphone advertisements or media applications, thereby gradually forming materialistic values (Dinh & Lee, 2022; J. Jiang et al., 2016b; Zawadzka et al., 2021). Similarly, cultivation theory (Gerbner, 1969) suggests that the exaggerated material wealth in the virtual world may foster a misperception of universal affluence among adolescents. Consequently, frequent exposure to such content can lead to the assimilation of materialistic values (Gerbner et al., 1982; Leyva, 2019; Moldes & Ku, 2020; Shrum et al., 2005; Sirgy et al., 1998; Wang et al., 2020a; Zhu et al., 2020).

Empirical studies also support this view. First, a “follow-up study” (Opree et al., 2014) and a quasi-experimental (Brand & Greenberg, 1994) and empirical research (Shrum et al., 2011) drew the conclusion that exposure to materialistic content disseminated through media or advertisements could lead to an increase in materialism. Considering the fact that smartphones have emerged as a significant medium for marketing and media applications in China (CNNIC, 2025), recent research has found that increases in media exposure result in a rise in materialistic values (Moldes et al., 2022), internet use is positively related to materialism (Zawadzka et al., 2022; Zhu et al., 2020), and excessive social network site usage has a predictive effect on materialism (Sharif & Khanekharab, 2017; Wang et al., 2020a; Yang et al., 2025). Apart from this, a cross-sectional study revealed that PSU exerts a positive predictive effect on adolescents’ materialism (Wang et al., 2020b). 

#### 2.1.2. Materialism as a Possible Antecedent of PSU

According to the self-escape theory of materialism (Baumeister, 1988, 1990; Baumeister & Scholar, 1991; Donnelly et al., 2016), materialistic individuals often cope with life dissatisfaction and negative emotions by entering a cognitively deconstructed state. This narrow, present-focused mindset can reduce inhibition, leading to impulsive behaviors such as PSU (Billieux, 2012; Longmire et al., 2021; Roberts & Pirog, 2013). Additionally, the uses and gratifications theory (L. Huang et al., 2014; Katz et al., 1973) posits that individuals use technology to fulfill specific psychological needs. For materialists, smartphones serve as effective platforms for social comparison and self-presentation, reinforcing the urge to use these devices to gratify their desire for material acquisition (Gentina & Rowe, 2020; Q. Li et al., 2017; Ouyang et al., 2020; Ozimek et al., 2017; Ozimek & Förster, 2017; Wolniewicz et al., 2018).

Empirical studies demonstrate the positive predictive effect of materialism on PSU among young adults (Lee et al., 2017; Roberts et al., 2015) and adolescents (Gentina & Rowe, 2020; Long et al., 2021; Ouyang et al., 2020). Studies have found that materialistic subjects are inclined to display social status through expensive possessions, while the smartphone itself, as a valuable item, is often regarded as a representative commodity that can display wealth to others. Therefore, highly materialistic individuals may particularly favor smartphone use and are more susceptible to developing PSU (Lee et al., 2018; Roberts & Pirog, 2013). Expanding on this susceptibility, recent empirical evidence suggests that materialism also drives maladaptive usage patterns beyond the device’s physical value. Specifically, Ozimek et al. (2024) demonstrated that materialists are prone to social media addiction—a core dimension of PSU—as they utilize these platforms to satisfy needs for social comparison and possession-defined success.

### 2.2. The Need and Significance of Utilizing RI-CLPM

The relationship between PSU and materialism involves two types of effects: (1) the between-person effects that reflect time-stable differences between individuals in the association of PSU and materialism and (2) the within-person effects that clarify how the fluctuation in PSU (or materialism) within an individual impacts materialism (or PSU). Both the between-person effects and within-person effects can be effective, and each has its unique theoretical and practical implications (Curran & Bauer, 2011). Between-person and within-person effects reflect different associations between variables, and studying the two effects separately in longitudinal studies has yielded completely different findings (Keijsers, 2016; Kojima et al., 2021; Takahashi et al., 2022; Zhou et al., 2020). Thus, studying only one level of the effects might hinder a more accurate and complete understanding of the dynamic associations between variables. Therefore, when examining the causal relationship between PSU and materialism, it is important to explore whether the association exists within a given individual or across different individuals.

The cross-lagged panel model (CLPM) stands as a prevalent approach for testing the potential reciprocal causal relationship among variables (Orth et al., 2021; Takahashi et al., 2022; Usami, 2021; Wu et al., 2013) However, in recent years, it has been criticized for not separating within- and between-person effects (Berry & Willoughby, 2017; Kojima et al., 2021; Lucas, 2023; Zhou et al., 2020). However, some studies have claimed that CLPM examines the predictive effects between variables based on between-person variance (Orth et al., 2021). Therefore, the interpretation of the prediction effect between variables analyzed by the CLPM remains a contentious issue in the field. The RI-CLPM includes random intercept factors that capture the stable trait-like between-person variance in each variable and obtain between-person effects of the construct based on a longitudinal correlation between variables (Hamaker et al., 2015; Orth et al., 2021; Usami, 2021). Consequently, the cross-lagged effect of RI-CLPM indicates a longitudinal within-person effect that tests whether a temporary within-person deviation from the trait level of one variable has a prospective effect on change in the temporary within-person deviation from the trait level of the other variable (Hamaker, 2023; Mulder & Hamaker, 2021; Orth et al., 2021).

### 2.3. The Current Study

In summary, it is possible that there is a negative cycle of PSU and materialism, and it is crucial to examine whether the association between the two variables exists within a given individual or across different individuals. This study utilized a longitudinal design with three waves of data to investigate the potential reciprocal associations between PSU and materialism. Both the CLPM and RI-CLPM were fit to the data, and longitudinal relationship between PSU and materialism were analyzed; the within-person and between-person effects in the association were also further disaggregated.

Based on previous research, we propose the following hypotheses:

**Hypothesis 1.**  *At the between-person level, PSU and materialism are expected to be positively correlated, indicating that individuals with higher levels of PSU relative to their peers will also report higher levels of materialism.*

**Hypothesis 2a.**  *(Use CLPM analysis.) PSU will positively predict subsequent materialism, and materialism will positively predict subsequent PSU.*

**Hypothesis 2b.**  *(Use RI-CLPM analysis.) The within-person cross-lagged paths between PSU and materialism will not be statistically significant. The observed longitudinal relationship is primarily attributable to time-invariant, trait-like factors rather than a dynamic, causal process at the within-person level.*

## 3. Materials and Methods

### 3.1. Participants and Procedure

Students in grades 3 to 10 from 12 randomly selected basic education schools in four cities of central China were assessed at three time points (April 2019, July 2020, and April 2021). A total of 3029 students (aged 7.92–17.88 years, mean age: 12.26 ± 2.36 years, boys: 50.00%) participated in our study. These age and gender characteristics were assessed at baseline (Wave 1, April 2019). Of the 3029 students, 3029 (100.00%), 2456 (81.08%), and 2699 (89.11%) participated at Waves 1, 2, and 3, respectively. All subjects participated in at least two wave measurements. The average missing rate for each variable was low (7.69% in total). Little’s MCAR test (Little, 1988) yielded a significant result (χ^2^ = 121.494, *df* = 55, *p* < 0.001), suggesting the data were not missing completely at random (MCAR). However, this significance is likely attributable to the large sample size (*N* = 3029), which renders the test overly sensitive to trivial deviations from randomness (Enders, 2010; Schafer & Graham, 2002). Consequently, we assumed the data to be missing at random (MAR), a condition under which the expectation–maximization (EM) algorithm yields unbiased parameter estimates and standard errors (Graham, 2009). Given the low percentage of missing data and the robustness of EM under the MAR assumption, missing values were handled using the EM algorithm.

Adolescents filled out the questionnaire independently at school. One parent of each adolescent reported information related to the family. In Wave 2, due to the influence of the COVID-19 pandemic, the test was conducted by sending the online questionnaire link to the participants via WeChat. Informed consent was obtained from teachers, school administrators, students, and their guardians for this study. This study was approved by the Institutional Review Board of the State Key Laboratory of Cognitive Neuroscience and Learning of Beijing Normal University.

### 3.2. Measures

#### 3.2.1. PSU in Waves 1 to 3

We used the modified Smartphone Addiction Proneness Scale (SAPS), which was developed by Kim (Kim et al., 2014). Although the scale is originally titled “addiction proneness”, it is widely used in the current literature to operationalize the construct of PSU (B. Li et al., 2023). And the reliability and validity are good when applied to Chinese adolescents (S. Huang et al., 2021). It includes four dimensions: disturbance of virtual life, virtual life orientation, withdrawal, and tolerance. Each dimension consists of four items, ranging from one (“strongly disagree”) to four (“strongly agree”). These scores add up to indicate the degree of PSU, with higher scores indicating a higher tendency of PSU. The Cronbach’s alpha is 0.902, 0.956, and 0.936 from Wave 1 to Wave 3, respectively.

#### 3.2.2. Materialism in Waves 1 to 3

Materialism was assessed with the Chinese version of the six-item Material Values Scale (Richins, 2004), which was revised by (W. Jiang et al., 2021). It has shown good reliability and validity in the sample of Chinese youth. The scale consists of three dimensions, namely (1) success, (2) centrality, and (3) happiness, which assesses the degree to which participants view the possession and acquisition of material goods as the key indicator of success, the primary life goal, and a pathway to achieving happiness. Each dimension contains two items that are evaluated using a four-point Likert scale from one (“does not match my experiences at all”) to four (“definitely matches my experiences”). Average scores were computed, with higher mean scores indicating higher levels of materialism. The Cronbach’s alpha values for the total scale were 0.765, 0.916, and 0.878 from Wave 1 to Wave 3, respectively.

#### 3.2.3. Demographic Information

We gathered students’ age, gender, grade, and socioeconomic status (SES) in Wave 1. Gender was coded as one for male and two for female. According to methods of previous studies (Y. Li et al., 2015), the standard scores of paternal education level, maternal education level, and annual household income in Wave 1 were averaged as an SES index, with higher scores reflecting higher SES. Among these demographic variables, age and SES were considered baseline covariates of the study due to their significant relationship with PSU and/or materialism, as evidenced in previous research (e.g., Busch & McCarthy, 2021; Chaplin & John, 2007; W. Jiang et al., 2021; Lai et al., 2022; Rindfleisch et al., 1997).

### 3.3. Statistical Analyses

Longitudinal associations between PSU and materialism across three time points were modeled into three models using structural equation modeling in Mplus 8.0 (Muthén & Muthén, 2017). First, we utilized a standard CLPM. Second, given that extended autoregressions (e.g., second-order autoregressions) of a CLPM can explain the stability of interindividual differences more comprehensively than the first-order autoregressions, which are necessary to obtain a CLPM to have an acceptable fit (Ehm et al., 2019; Mulder & Hamaker, 2021), we further constructed a CLPM with another two second-order autoregressive paths for PSU and materialism between Wave 1 and Wave 3. Third, we used the RI-CLPM which could be attributed to within-person fluctuations over time and between-person differences, respectively (Hamaker et al., 2015; Orth et al., 2021). We also used the CLPMs to clearly demonstrate a comparison of the different outcomes of the CLPMs and the RI-CLPM.

## 4. Results

### 4.1. Descriptive Statistics and Bivariate Correlations

Correlation analyses indicated that the correlations between PSU and materialism at T1, T2, and T3 were significantly positive (see Table 1).

Correlation analyses revealed significantly positive associations between PSU and materialism at all waves (T1: r = 0.45, *p* < 0.01; T2: r = 0.45, *p* < 0.01; T3: r = 0.51, *p* < 0.01), indicating stable between-wave relationships. The means for PSU ranged from 1.87 to 1.97 across waves, while materialism scores varied from 1.76 to 2.02, suggesting minor fluctuations over time. These patterns underscore the persistent covariation between constructs, supporting further longitudinal modeling.

### 4.2. Standard Cross-Lagged Panel Model

**Figure 1:**
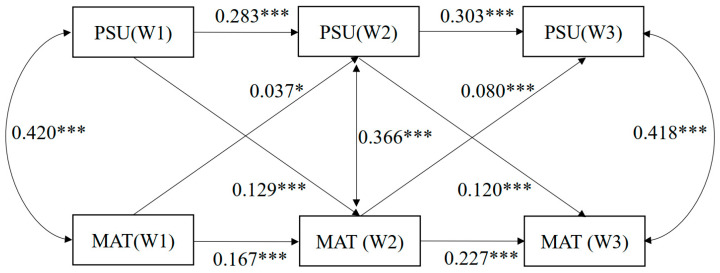
Standardized regression coefficients in the CLPM with first-order autoregressions. Note: *p* value significant at * *p* < 0.05, *** *p* < 0.001. For the clarity of the presentation, pathways that include covariates and their respective coefficients are omitted, and non-significant paths between covariates and PSU/materialism were deleted in the statistical analysis for a better model fit. Abbreviations: PSU, problematic smartphone use; MAT, materialism.

The CLPM did not fit the data well, wherein χ^2^(6) = 460.148, *p* < 0.001, RMSEA = 0.158, with 90% CI [0.146, 0.170], CFI = 0.908, TLI = 0.587, SRMR = 0.060 (see Table 2 below). The standardized regression coefficients between all variables shown in Figure 1 were significant. More precisely, all autoregressive stability paths in the model were positive and significant. The information from the cross-lagged pathways clearly supported the deduction of mutual relationships. That is, PSU was positively associated with the subsequent materialism and materialism was also positively associated with the subsequent PSU across three waves. After controlling the age and SES, results were robust.

### 4.3. Cross-Lagged Panel Model with Second-Order Autoregressions

**Figure 2:**
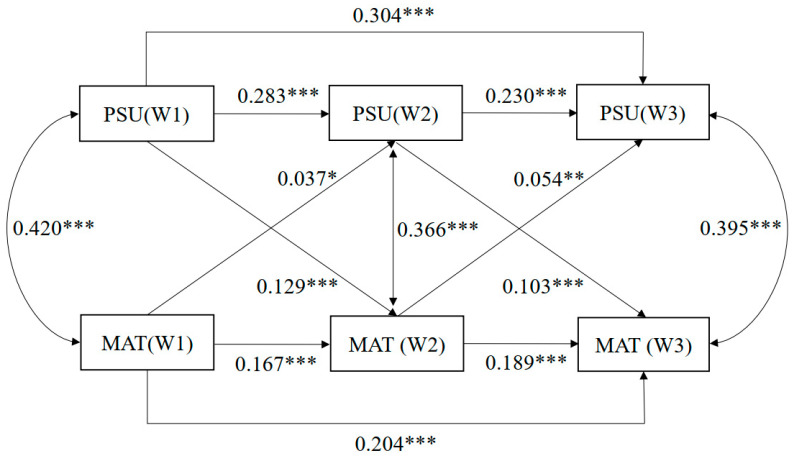
Standardized regression coefficients in the cross-lagged panel model with second-order autoregressions. Note: *p* value significant at * *p* < 0.05, ** *p* < 0.01, *** *p* < 0.001. Non-significant paths between covariates and PSU/materialism were deleted in the statistical analysis for a better model fit. Abbreviations: PSU, problematic smartphone use; MAT, materialism.

Wave 1 levels of each construct may directly predict Wave 3 levels, independent of Wave 2. This modeling choice is also conceptually aligned with the theoretical understanding of trait-like stability in both PSU and materialism. Both constructs are likely influenced by enduring individual predispositions. Inclusion of the two second-order autoregressive paths between Wave 1 and Wave 3 for PSU and materialism did not change the cross-lagged pathways (see Figure 2, the direction and significance of the relationships between PSU and materialism were almost the same as the standard CLPM) but greatly improved the model fit; (∆χ^2^(1) = 432.438, *p* < 0.001, RMSEA = 0.039, with 90% CI [0.025, 0.053], CFI = 0.995, TLI = 0.975, SRMR = 0.015) (see Table 2). 

### 4.4. RI-CLPM

**Figure 3:**
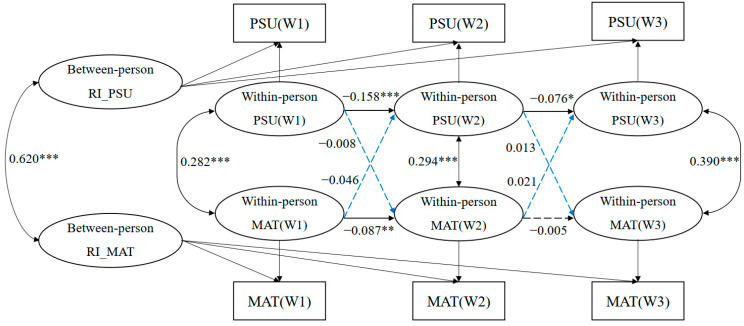
Standardized regression coefficients in the random intercept cross-lagged panel model. Note: *p* value significant at * *p* < 0.05, ** *p* < 0.01, *** *p* < 0.001. Blue lines represent cross-lagged associations, while black lines represent autoregressive stability paths and correlations. Solid lines indicate statistically significant paths (*p* < 0.05), whereas dashed lines indicate non-significant paths (*p* > 0.05). Non-significant paths between covariates and PSU/materialism were deleted in the statistical analysis for a better model fit. Abbreviations: PSU, problematic smartphone use; MAT, materialism; RI_PSU, random intercept of problematic smartphone use; RI_MAT, random intercept of materialism.

To further disentangle whether the above associations between PSU and materialism found in the CLPMs were driven by individual, within-person developmental processes or by between-person differences, we fit an RI-CLPM that displayed good model fit (see Figure 3), with χ^2^(10) = 220.412, *p* < 0.001, RMSEA = 0.083 with 90% CI [0.074, 0.093], CFI = 0.958, TLI = 0.885, SRMR = 0.048, and was better fit than the standard CLPM with first-order autoregressive paths (∆χ^2^(4) = 239.736, *p* < 0.001) (see Table 2). The random intercepts of PSU and materialism were significantly associated (*r* = 0.620, *p* < 0.001) at the between-person level. However, there was no significant cross-lagged association between the two variables over time at the within-person level. Autoregressive stability paths were negative and significant across both time lags for PSU (*β* = −0.158, *p* < 0.001; *β* = −0.076, *p* < 0.05) and from Wave 1 to Wave 2 for materialism (*β* = −0.087, *p* < 0.01). However, the autoregressive path from Wave 2 to Wave 3 for materialism was not significant (*β* = −0.005, *p* > 0.05). This suggested that although adolescents with higher PSU had higher levels of materialism (between-person effects), their own individualized fluctuations in PSU did not covary with fluctuations in materialism (within-person effects). 

The RI-CLPM demonstrated a better fit than the standard CLPM, as evidenced by lower RMSEA (0.083) and higher CFI (0.958). This supports the utility of separating stable between-person differences from within-person fluctuations, aligning with contemporary longitudinal methodologies (Hamaker et al., 2015).

## 5. Discussion

Our study is the first to test the temporal directional association between PSU and materialism, and it separates the within- and between-person effects. Our main findings revealed that PSU and materialism were mutually linked over time at the between-person level, while there was no significant cross-lagged association between the two variables at the within-person level. Our findings highlight the necessity to disaggregate within-person and between-person effects and to understand the pattern of their dynamic associations.

### 5.1. The General Dynamic Associations Between PSU and Materialism: Evidence from CLPMs

The results of the standard CLPM demonstrated a reciprocal positive association between PSU and materialism consistently across waves, although its model fit was insufficient. With the inclusion of the Wave 1 to Wave 3 autoregressive paths, the direction and significance of the relationship between PSU and materialism were almost the same, while the model fit was greatly improved. Thus, on one hand, the two second-order autoregressive paths may conceptually imply some form of “sleeper” effect in variable stability (i.e., temporal stability) where the Wave 1 level of each variable predicted change in its Wave 3 level but not (entirely) through affecting its level at Wave 2 (Rote et al., 2020). On the other hand, it could be the stable individual differences at the overall level of each variable (i.e., time-invariant, trait-like stability) that led to this result (Lüdtke & Robitzsch, 2022), which was then specifically studied through the random intercepts in the RI-CLPM. 

The existence of this possibility could verify that the long-run, trait-like stability of interindividual differences may indeed exist in PSU and materialism, which were captured more comprehensively by the second-order lagged relations. Thus, this finding shed further light on the importance of utilizing RI-CLPM to disaggregate stable between-person differences from within-person fluctuations over time to see the nature of the longitudinal association between PSU and materialism.

Although there were differences in the model fit of the two CLPMs, the direction and significance of the cross-lagged effect were almost the same, suggesting that the reciprocal relation of PSU and materialism was robust when within-person effects and between-person effects were not distinguished. Statistically, these results indicate that adolescents with high PSU relative to their peers are more likely to experience a subsequent rank-order increase in materialism compared to those with low PSU and vice versa. Even though the theoretical interpretation of CLPM cross-lagged effects (e.g., whether they are between-person effects or uninterpretable blend effects) is still a question of methodological debate (Berry & Willoughby, 2017; Lucas, 2023; Masselink et al., 2018; Mastrotheodoros et al., 2020), the findings do help to clarify extant cross-sectional research on the association between PSU and materialism (Long et al., 2021; Wang et al., 2020b; Yang et al., 2024) by utilizing the standard approach to investigate the question of temporal ordering. Crucially, while previous studies primarily supported a bidirectional “vicious cycle” hypothesis between materialism and PSU (Gentina & Rowe, 2020; Wang et al., 2020b), our findings offer a more nuanced perspective. By contrasting the results of CLPM and RI-CLPM, we demonstrated that this apparent “cycle” is largely driven by stable between-person differences rather than immediate within-person contagion. This distinction helps reconcile inconsistencies in the literature, suggesting that prior findings of reciprocal causality may have overestimated the dynamic interplay due to the confounding influence of stable individual traits.

### 5.2. Disaggregated Within- and Between-Person Effects Between PSU and Materialism: Evidence from RI-CLPM

The absence of within-person effects in the RI-CLPM suggests that theories such as social learning theory and the self-escape theory of materialism may be more applicable at the between-person level. That is, individuals who are chronically higher in neuroticism or chronic stress (stable traits) may be more likely to both overuse smartphones and endorse materialistic values, rather than these constructs dynamically influencing each other within the same person over time.

The between-person results converge with prior research to indicate which members of our sample are higher in materialism: those who have a higher level of PSU and vice versa (Ouyang et al., 2020; Wang et al., 2020b). However, within-person results showed that adolescents’ fluctuations in PSU beyond their typical levels held no predictive power over their future changes in materialism, and vice versa. The results may suggest that the reciprocal associations between PSU and materialism detected in previous studies and found in the CLPMs of the present study might be accounted for stable between-person differences as common causes in the longitudinal data over time, such as stable personal traits and chronic stressors. For example, as individuals with the personality trait of neuroticism have high social anxiety in in-person settings, they are more likely to seek social comfort through smartphone-mediated communication, and they also tend to seek compensation for the need of security and belongingness through materialistic ways (Busch & McCarthy, 2021; Chang & Arkin, 2002; Gao et al., 2017; Horwood & Anglim, 2018; Kasser, 2016). Likewise, life stress may not only motivate people to overuse smartphones as a means of alleviating their negative emotions, but may also lead people to materialism since they adopt the search for material goods as a strategy to cope with stress (Kardefelt-Winther, 2014; Moschis, 2007; Walburg et al., 2016; Weaver et al., 2011). These findings might offer a new perspective for interventions into adolescents’ PSU and materialism by ameliorating the common causes, such as the neurotic personality and stress factors. Further studies can explore these factors more accurately or pinpoint stable or enduring factors that would interrupt the unyielding cycle of PSU and materialism. Besides the stable trait-like factors, there are alternative explanations for the non-significant within-person effects. One plausible explanation concerns the time interval of measurement. The interplay between materialism and PSU might operate on a shorter time scale (e.g., days or weeks) rather than the one-year interval adopted in this study. For instance, the “escapism” mechanism—where individuals use smartphones or material acquisition to cope with negative emotions—often occurs as an immediate response to situational stressors (Panova & Carbonell, 2018). Such transient, short-term fluctuations might neutralize over the course of a year, making them undetectable in annual wave data (Keijsers & Van Roekel, 2019). Additionally, the absence of within-person effects could suggest that the reciprocal relationship is not universal but context-dependent. It may only manifest under specific conditions, such as during periods of significant life transitions or high academic pressure, which might not have been captured uniformly across the entire sample.

The result indicates that the dynamic association between PSU and materialism is not a within-person process. Conversely, the existence of significant cross-lagged effects in the CLPMs could suggest that the reciprocal relationship might be a between-person issue (Orth et al., 2021), although the comprehension of the statistical effects remains a contentious issue (Berry & Willoughby, 2017; Keijsers, 2016; Mastrotheodoros et al., 2020). That is, it appears that the relative status of adolescents’ PSU compared to their peers is crucial for understanding who is more likely to develop materialism, and vice versa. Theoretically, these results may provide preliminary verification that the existing theoretical hypotheses (i.e., social learning theory; (Bandura, 1969); cultivation theory; (Gerbner, 1969); self-escape theory of materialism; (Donnelly et al., 2016); uses and gratifications theory; (Katz et al., 1973)) are more applicable to explain the links between PSU and materialism at the between-person level than at the within-person level. Further empirical studies and theoretical refinement may be necessary to verify the null findings at the within-person level of the RI-CLPM and to understand where the reciprocal associations between PSU and materialism would take place on the ecological and time-scale level.

Furthermore, considering the cultural context is vital for interpreting these findings. Unlike Western individualistic cultures where materialism often serves as self-expression, in collectivist societies, materialism is frequently linked to social conformity and the concept of “Mianzi” (face)—the need to maintain social status within the group (Liao & Wang, 2009). Consequently, adolescents with higher materialism may be more prone to PSU as a means to engage in social comparison and seek validation on social media, reinforcing the stable between-person differences observed in our study. Additionally, adolescents face exceptionally high academic pressure (Zhao et al., 2015), in such high-stakes environment, smartphones may serve a unique function as a stress-coping mechanism (i.e., escapism). The cultural and educational context highlights the importance of investigating the materialism–PSU link in China, as the underlying motivations for these behaviors may differ from those in Western populations.

### 5.3. Implications, Limitations, and Future Directions

As for the contributions, we conducted both CLPM and RI-CLPM analyses to investigate the longitudinal relationship between PSU and materialism for the first time. Comparing results of the two models provides an important suggestion to separate within-person and between-person effects to understand the temporal directional relationship between PSU and materialism. The main findings indicate that the bidirectional linkages between PSU and materialism that were previously detected and found in our CLPM analysis may result from unobserved stable between-person differences as common causes rather than being indicative of causal effects. Beyond the methodological application of the RI-CLPM, this study makes a unique theoretical contribution by challenging the prevailing causal narrative in the field. Our null findings at the within-person level suggest that materialism and PSU may not directly cause each other over time but rather co-occur as manifestations of shared underlying vulnerabilities (e.g., personality traits or environmental factors). This shifts the theoretical focus from intervening in the “cycle” to addressing the root causes that sustain both high materialism and PSU, providing a new framework for future research to identify these common antecedents. From a practical perspective, the significant between-person effects might also provide valuable insight for detecting and ameliorating adolescents’ PSU and materialism. That is, adolescents with PSU levels higher than their peers tend to also experience higher levels of materialism and vice versa. Thus, the results indicate that it is possible to identify which adolescents might be more in need of intervention compared with their peers (Masselink et al., 2018; Mastrotheodoros et al., 2020). It inspires us to develop interventions aimed at promoting healthier consumption patterns among adolescents.

Apart from the contributions, there are also some limitations. First, we gathered data on the main research variables from adolescents through self-reporting, which could potentially be influenced by social desirability. Further research can use the multi-informative method to collect data, such as incorporating other reported data and/or implementing experimental designs, which would be essential to provide more robust insights. Second, despite the statistical rationale for using EM imputation, the significant MCAR test suggests a potential limitation. Future studies could employ more robust methods, such as Full Information Maximum Likelihood (FIML) to better account for missingness mechanisms. Third, our study focuses on four central Chinese cities, and it may be different across different cultures. Examinations of regional/cultural differences are important extensions of the present work that is left for future research. Fourth, the time intervals may also have drawbacks because the causal processes may occur at shorter time intervals that could not be captured in our study (Boer et al., 2021; Keijsers & Van Roekel, 2019; Orben, 2020). Addressing this issue requires the use of advanced approaches such as dynamic structural equation modeling (Asparouhov et al., 2018) and innovative data collection methods like experience sampling (Keijsers & Van Roekel, 2019) might be essential in future investigations to verify our study results. Subsequent studies should aim to include measures of core personality traits, mental health indicators, and detailed social environmental factors to clarify whether the PSU–materialism relationship is mediated or moderated by these variables. Furthermore, while the current study provides important insights into the overall adolescent population, future research should examine developmental differences by comparing distinct age groups. Such investigations could reveal whether the null within-person effects and significant between-person associations observed in our study vary across developmental stages, potentially informing more targeted intervention strategies.

Finally, our research indicates that the longitudinal association between PSU and materialism might be interpreted by between-person differences as common causes (e.g., neurotic personality, family stressors). Future research can further specify the persistent factors, explaining the between-person effects in the association that should be addressed to disrupt the PSU–materialism cycle. Additional investigation is required to more precisely identify the long-term or stable factors that could explain the observed between-person effects in the association, with the aim of disrupting the detrimental cycle of PSU and materialism. Apart from these, future studies should test the cross-cultural generalizability of these effects, particularly in individualistic societies. Research could also examine how specific social media platforms and consumption environments moderate these associations.

### 5.4. Practical Implications

Our findings offer several practical implications for addressing adolescent PSU and materialism. First, the significant between-person association provides a clear rationale for implementing comparative screening in school settings to identify at-risk adolescents who exhibit higher levels of both PSU and materialism relative to their peers, enabling targeted interventions. Crucially, the null within-person effects indicate that such interventions should address underlying, stable vulnerabilities (e.g., personality traits, chronic stress) common to both conditions rather than focusing solely on symptomatic behavior change. For parents and educators, these findings offer valuable guidance, suggesting that attention should be directed toward adolescents showing persistently high levels relative to peers, while understanding that occasional fluctuations fall within normal developmental ranges.

## 6. Conclusions

Utilizing the CLPM and the RI-CLPM, this study represents the first examination of the longitudinal associations between PSU and materialism and the first attempt to disentangle the within-person and between-person effects in the two-level associations. Results suggest that the reciprocal positive associations between PSU and materialism that were previously detected and found in the CLPMs of the present study may be attributable to confounding within-person effects with between-person effects rather than being indicative of causal effects. 

These findings enrich our comprehension of the dynamic links between PSU and materialism and emphasize the necessity of separating within- and between-person effects to clarify the longitudinal associations. From a practical perspective, our findings indicate that adolescents with higher PSU levels compared to their peers tend to experience higher levels of materialism and vice versa, thus indicating which adolescents need an intervention. Future studies can explore some common causes underlying the between-person associations of the two constructs.

## Figures and Tables

**Table 1:** Descriptive statistics and bivariate interrelations.

Variable	1	2	3	4	5	6	7	8
1. W1 PSU	—							
2. W2 PSU	0.37 **	—						
3. W3 PSU	0.48 **	0.41 **	—					
4. W1 Materialism	0.45 **	0.20 **	0.27 **	—				
5. W2 Materialism	0.27 **	0.45 **	0.27 **	0.25 **	—			
6. W3 Materialism	0.29 **	0.28 **	0.51 **	0.32 **	0.33 **	—		
7. Teen age	0.39 **	0.32 **	0.34 **	0.16 **	0.24 **	0.28 **	—	
8. Teen SES	−0.20 **	−0.06 **	−0.18 **	−0.07 **	−0.07 **	−0.10 **	−0.25 **	—
*M*	1.87	1.97	1.94	1.76	2.02	1.86	12.26	−0.02
*SD*	0.62	0.63	0.63	0.61	0.64	0.65	2.36	0.80

Note: *p* value significant at ** *p* < 0.01. Abbreviations: W1–W3, Waves 1–3; PSU, problematic smartphone use; SES, socioeconomic status; M, means; SD, standard deviations.

**Table 2:** Model fit statistics for model comparison of associations between PSU and materialism.

	χ^2^	*df*	CFI	TLI	SRMR	RMSEA	∆χ^2^	∆*df*	*p* Value ^†^
CLPM1	460.148	6	0.908	0.587	0.060	0.158	—	—	—
CLPM2	27.710	5	0.995	0.975	0.015	0.039	432.438	1	<0.001
RI-CLPM	220.412	10	0.958	0.885	0.048	0.083	239.736	4	<0.001

Note: ∆χ^2^ and ∆*df* of CLPM2 and RI-CLPM are the results of comparison with CLPM1; ^†^ likelihood ratio test. Abbreviations: CLPM1, cross-lagged panel model with first-order autoregressions; CLPM2, cross-lagged panel model with second-order autoregressions; RI-CLPM, random intercept cross-lagged panel model; CFI, comparative fit index; SRMR, standardized root mean square residual; TLI, Tucker-Lewis index; RMSEA, root mean square error of approximation.

## Data Availability

The data that support the findings of this study are available from the corresponding author upon reasonable request.

## Abbreviations

The following abbreviations are used in this manuscript:

CLPM	Cross-Lagged Panel Model
MAT	Materialism
PSU	Problematic Smartphone Use
RI-CLPM	Random Intercept Cross-Lagged Panel Model
SES	Socioeconomic Status
FIML	Full Information Maximum Likelihood
